# Seroepidemiology of SARS-CoV-2, Yamagata, Japan, June 2020

**DOI:** 10.5365/wpsar.2020.11.3.012

**Published:** 2021-02-01

**Authors:** Keita Morikane, Naohito Satoh, Kanji Hatano, Kazunori Kanouchi, Seiji Kakehata, Shinya Satoh, Timothy M. Uyeki, Yoshiyuki Ueno

**Affiliations:** a Yamagata University, Yamagata, Japan.; b Centers for Disease Control and Prevention, Atlanta, Georgia, United States of America.

In Japan, the first case of coronavirus disease 2019 (COVID-19) was identified in mid-January 2020, and cases peaked in the spring at 720 cases per day on 11 April. Thereafter, the number of reported cases per day declined to 50 on 15 May and remained low until mid-June, when numbers again started to increase. On 5 August, 1234 cases were reported, giving a cumulative total of 40 485 cases, with a case fatality proportion of 2.5% (1021 deaths). (*1*) Although COVID-19 is designated as a reportable disease in Japan, severe acute respiratory syndrome coronavirus 2 (SARS-CoV-2) testing capacity was limited in the early stage of the pandemic. It took up to 4 days for specimens to be tested by reverse transcription polymerase chain reaction (RT–PCR). The Japanese Government recommended that anyone with mild illness symptoms should stay at home, to avoid overwhelming health-care facilities. SARS-CoV-2 testing was prioritized for hospitalized patients and those with chronic comorbidities. Thus, the true number of symptomatic cases of COVID-19 in Japan is likely to be far greater than the number of reported cases.

In one Chinese study, SARS-CoV-2-specific immunoglobulin IgG and IgM were detected in serum samples from most patients (asymptomatic or symptomatic) who were diagnosed with SARS-CoV-2 by RT–PCR. (*2*) This finding implies that seroepidemiological studies can be used to estimate the infection rate of SARS-CoV-2 in a population. Estimating the point prevalence of SARS-CoV-2 infections might be helpful in assessing population susceptibility, and in balancing public health control measures with the reopening of social and economic activities. Results from several seroepidemiological studies have been published, with seroprevalence reported from Spain (5%), Switzerland (10.8%) and the United States of America (1–6.9%, 4.65% and 14%). (*3*-*7*) These studies were performed in countries where the incidence of COVID-19 was high. In countries in the Asia-Pacific, where COVID-19 incidence was low, a few SARS-CoV-2 seroepidemiology studies have been conducted that are not population based. Among these studies, seroprevalence was 7.6% from a single-centre study of outpatients and their guardians in the Republic of Korea, and 0.4% in a study using residual sera collected at a single hospital in Malaysia. (*8*, *9*)

We conducted a cross-sectional seroepidemiological study in Yamagata Prefecture, an urban–rural area in northern Japan, where the incidence of reported COVID-19 cases was 0.007% (i.e. 76 cases among a population of about 1.07 million, as of 5 August 2020). (*1*) This is lower than the overall incidence of COVID-19 cases reported throughout Japan (0.034%), and lower than in most Japanese prefectures and the Tokyo metropolitan area (0.102%); however, it is higher than in some low-incidence prefectures (0–0.002%). (*1*) Residual sera obtained from patients who visited the outpatient clinic of Yamagata University Hospital for any acute medical condition during 1–4 June 2020 were tested for SARS-CoV-2 antibody. Blood samples were collected for clinical diagnostic purposes and, after use, were de-identified before serological testing was performed. Because samples were de-identified, individual consent was not obtained. This study was approved by the Ethics Committee of Yamagata University School of Medicine.

Serological testing was performed using an electrochemiluminescence immunoassay (ECLIA) Elecsys® Anti-SARS-CoV-2 on Cobas® e601 module (Roche Diagnostics, Basel, Switzerland). This qualitative assay detects total antibody – primarily IgG, but also IgM and IgA antibody – to the nucleocapsid protein of SARS-CoV-2. A cut-off optical density (OD) index value of 1.0 was used to define a seropositive result. According to the manufacturer’s fact sheet, the specificity of the serological assay is 99.80% (i.e. 21 false positives among the 10 453 specimens collected before December 2019). (*10*)

Among 1009 samples tested, five specimens were positive for SARS-CoV-2 antibody. The estimated seroprevalence of SARS-CoV-2 infections was 0.50% (95% confidence interval [CI]: 0.062–0.93%). The OD values of five seropositive specimens varied substantially; two had OD values close to the cut-off index value (1.3 and 1.6), suggesting low antibody titres, and three were above 5.0. Using the 95% CI for the seroprevalence estimate of 0.50%, we estimated that the Yamagata Prefecture population had 670–10 000 SARS-CoV-2 antibody-positive individuals.

Our study has several limitations. First, sera used in this study were obtained from patients visiting our hospital’s outpatient acute care clinic; hence, this sample is probably not representative of the general population of Yamagata Prefecture. Also, because the serum specimens were de-identified, we did not have any demographic data to determine representation across age groups. Second, the specificity of the assay suggests an anticipated false positive rate of 0.20%, which may affect the reliability of the estimated seroprevalence in our study. Third, in a population with a low prevalence of SARS-CoV-2 infections, as was the case in Yamagata, false positives are more likely than in a population with high prevalence. Slight modification of the assay seropositive cut-off index value (e.g. from 1.0 to 1.6) would reduce the estimated seroprevalence. For example, if only the three strongly positive serum samples were considered to be true seropositive results, the estimated seroprevalence would be 0.30% (95% CI: 0–0.63%).

This cross-sectional seroepidemiological study in Yamagata Prefecture, Japan, identified low seroprevalence of SARS-CoV-2 antibody, suggesting that the population is highly susceptible to SARS-CoV-2. Additional studies with population-based sampling are needed to assess the impact of SARS-CoV-2 in this population over time.

## Ethical statement

Because samples were de-identified, individual consent was not obtained. This study was approved by the Ethics Committee of Yamagata University School of Medicine.
